# Generation and utilization of endostatin‐sensitive cell lines for assessing the biological activity of endostatin

**DOI:** 10.1002/mco2.506

**Published:** 2024-03-23

**Authors:** Xi Qin, Yifang An, Xiang Li, Fang Huang, Yong Zhou, Dening Pei, Hua Bi, Xinchang Shi, Wenhong Fan, Youxue Ding, Shuang Li, Shanhu Li, Junzhi Wang

**Affiliations:** ^1^ National Institutes for Food and Drug Control Beijing China; ^2^ WHO Collaboration Centre for Biologicals Standardization and Evaluation Beijing China; ^3^ Department of Cell Engineering Beijing Institute of Biotechnology Beijing China; ^4^ Sinovac Research & Development Co., Ltd. Beijing China

**Keywords:** biological activity assay, CRISPR/Cas9, endostatin, gene knockdown, HUVEC, recombinant protein

## Abstract

Recombinant proteins are gaining increasing popularity for treating human diseases. The clinical effectiveness of recombinant proteins is directly related to their biological activity, which is an important indicator in drug development and quality control. However, certain recombinant proteins have unclear or complex signal pathways, making detecting their activity in vitro difficult. For instance, recombinant human endostatin (endostatin), a new antitumor drug developed in China, lacks a sensitive and stable assay for its biological activity since being market approval. To address this issue, we performed a genome‐wide screening of immortalized human umbilical vein endothelial cells (HUVECs) using a CRISPR/Cas9 knockout library containing 20,000 targeted genes. We identified two potential endostatin‐resistant genes, NEPSPP and UTS2, and successfully constructed a highly sensitive cell line, HUVEC‐UTS2‐3#, by knocking down the UTS2 gene. Based on the optimized parameters of HUVEC‐UTS2‐3# cells, we established a new method for detecting the biological activity of endostatin. The method was validated, and it produced results consistent with primary HUVEC cells but with higher sensitivity and more stable data. The use of gene‐editing technology provides a novel solution for detecting the biological activity of recombinant proteins that other methods cannot detect.

## Graphical abstract

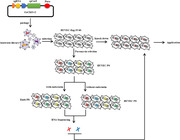

## 1 INTRODUCTION

The biological activity of recombinant proteins is directly related to their clinical effectiveness as drugs, playing an essential role in their development and quality control. Establishing the activity detection methods will boost the industrialization process of recombinant protein drugs. Various activity detection methods are adopted for different recombinant proteins. Due to simplicity, reliability, and high efficiency, transgenic cell lines^1^, ^2^ have been widely used to determine the biological activity assays of recombinant proteins, such as interferon α,^3^ keratinocyte growth factor,^4^ erythropoietin,^5^ and even some antibodies.^6^, ^7^ All of the developed methods rely on a single signal pathway that has been extensively studied; however, an effective approach is yet unavailable to determine in vitro activity of recombinant proteins involved in complex or unknown signaling pathways.

As early as the 1970s, Prof. Folkman from Harvard Medical School proposed that blocking the growth of blood vessels^8^ could halt the regular nutrient supply to tumor cells, thus inducing tumor cell apoptosis, and since then, the strategy of targeting tumor neovascularization has been commenced for cancer treatment.^9^ Recombinant human endostatin (trade name: Endostar, code: YH‐16), henceforth referred to as endostatin, was developed and launched in 2006 in China as a new antitumor drug.^10^ Endostatin participates in multiple signaling pathways and targets various signaling molecules, receptors, growth factors, and enzymes.^11^, ^12^, ^13^ It has low drug resistance, mild toxicity, and good patient compliance. Moreover, in comparison to other antiangiogenic‐targeted drugs, such as monoclonal antibodies or tyrosine kinase inhibitors, endostatin is more suitable for long‐term antitumor treatment and more effective prevention of tumor recurrence and invasion.^11^, ^12^, ^13^, ^14^, ^15^ Since listing, it has been applied in the treatment of various solid tumors in clinical practice, such as lung cancer,^16^ malignant melanoma,^17^ osteosarcoma,^18^ and nasopharyngeal carcinoma.^19^

Endostatin lacks an efficient and stable method for detecting its biological activity in vitro. Although endostatin has demonstrated promising clinical effects, its cellular mechanism remains unclear, hampering the establishment of a reporter gene method for measuring its biological activity in vitro. At present, primary human umbilical vein endothelial cell (HUVEC) cell inhibition is employed as a temporary method to measure endostatin's biological activity, but this method has a certain limitation in measuring a distinct inhibitory effect. The minimum effective concentration of endostatin is approximately 1 mg/mL, and the finished drug product (5 mg/mL injection) cannot be prediluted before detection. This makes it impossible to judge whether the cell death is caused by the drug's high concentration or its inhibitory effect on cell growth, leading to unreliable test results. Additionally, because HUVEC cells are primary cells and are limited to 10 generations for culture, they are not suitable for the activity detection of recombinant proteins.

In order to develop a cell line for the determination of the biological activity of recombinant proteins in the absence of clear signaling pathways, we performed genome‐wide screening of the immortalized HUVEC cells using a CRISPR/Cas9 knockout library, which is a powerful tool for high‐throughput genomic screening in cells.^20^, ^21^, ^22^, ^23^, ^24^, ^25^

In this study, two potential endostatin‐resistant genes were screened from a library containing 20,000 genes using CRISPR/Cas9 gene‐editing technology, and a drug‐sensitive cell line HUVEC‐UTS2‐3# was successfully constructed by knocking down the expression of UTS2, and demonstrated a good dose–response curve for endostatin. Based on the sensitivity of the constructed cell line, a new method was established for determining endostatin's biological activity. The established method successfully met the specificity, accuracy, precision, and stability requirements for detecting the biological activity as per the International Conference on Harmonisation Q2 (R2) guidelines, the American Association of Pharmaceutical Scientists/Food and Drug Administration (AAPS/FDA) Bioanalysis Symposium, and the provisions of the Chinese Pharmacopoeia (Volume III). The comparison with existing method showed a comparable consistency, but the established method presented more sensitivity and stability for detecting endostatin's biological activity, indicating its potential to replace the current methods for determining the biological activity of endostatin in vitro.

## 2 RESULTS

### 2.1 Establishment of the immortalized HUVEC cells

**FIGURE 1:**
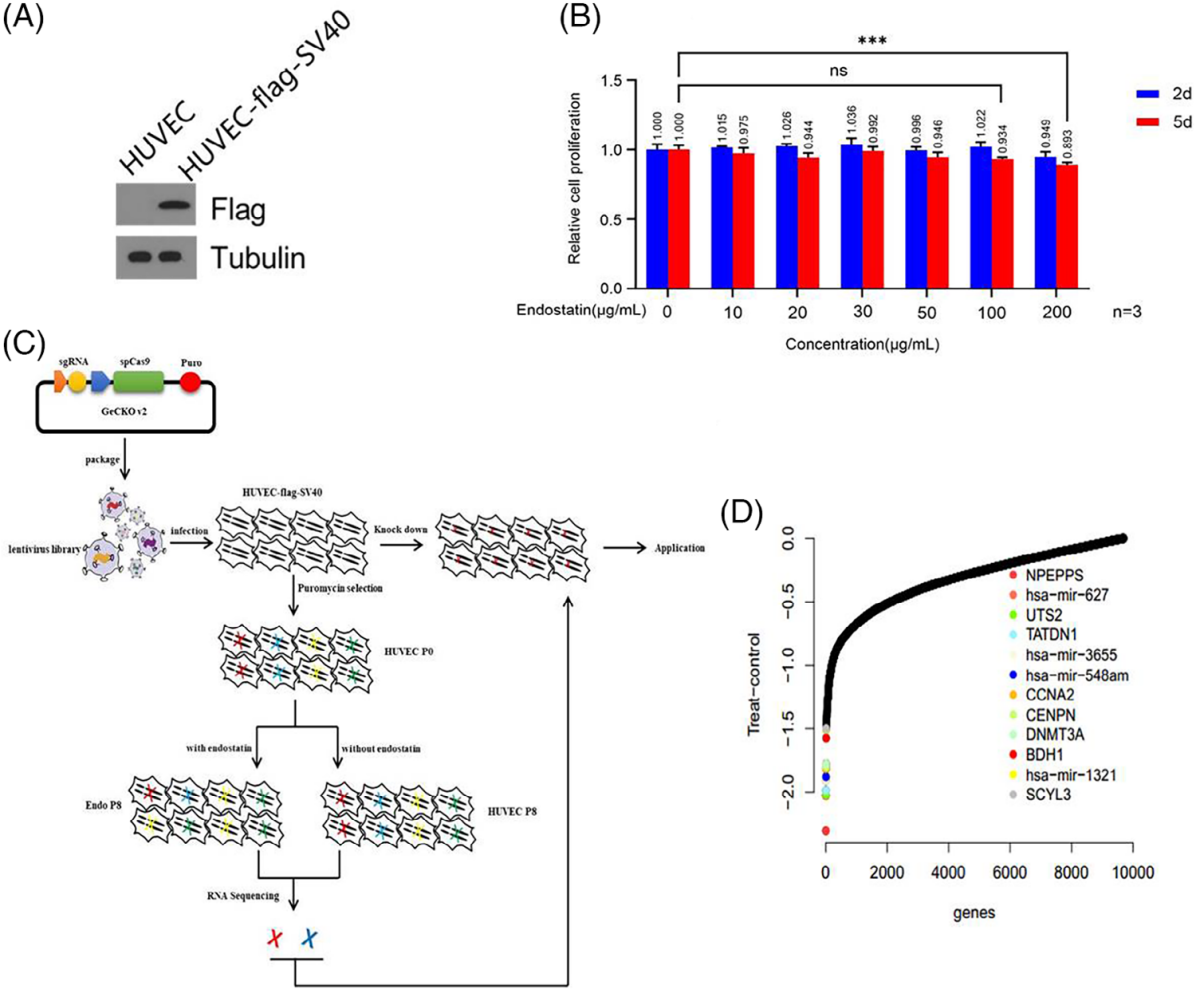
Results of potential endostatin‐resistant gene screening by CRISPR/Cas9 gene knockout libraries. (**A**) Western blot–detected expression of large T antigen in the immortalized human umbilical vein endothelial cell (HUVEC) cells using tubulin as the control protein. Antiflag antibody was used to detect large T antigen, large T antigen was constructed on a eukaryotic vector containing *flag*. (**B**) The HUVEC‐flag‐SV40 proliferation activity at different endostatin concentrations for 2 and 5 days was detected by CCK‐8 (ns, no significant difference; ***p < 0.001). (**C**) Screening process for endostatin resistance genes. sgRNAs were delivered into immortalized HUVEC cells (HUVEC‐flag‐SV40) by lentiviral infection. Under puromycin pressure, the cells were further cultured with/without endostatin for eight generations. RNA sequencing was performed on the zeroth‐ and eighth‐generation cells, respectively. The genes with significantly decreased were selected (UTS2 and NPEPPS), and their expression in HUVEC‐flag‐SV40 cells was downregulated by siRNA, respectively. The red “X” represents UTS2 gene. The blue “X” represents NPEPPS gene. (**D**) The sgRNA enrichment between the dosing group and the control group was analyzed by RNA‐seq, and the corresponding gene loci were assigned. The lower ratio of the dosing group to the control group indicated that the gene deletion increased the cell sensitivity to endostatin.

The inability of primary HUVEC cell line to proliferate beyond 10 passages makes it unsuitable for gene‐editing study. In the present study, after the primary HUVEC cells were infected with the packaged SV40 lentivirus in vitro, monoclonal clones were selected, screened in culture medium containing puromycin, and passaged to the 10th generation. The immortalized HUVEC cells survived and expressed Western blot‐detectable large T antigen in the 20th‐generation cells (Figure 1A). The immortalized cell line was successfully established and named HUVEC‐flag‐SV40. The concentration screening revealed inhibition of HUVEC‐flag‐SV40 cell proliferation at 100 μg/mL endostatin (Figure 1B). Therefore, this concentration was subsequently used for pressure enrichment.

### 2.2 The screening of potential endostatin‐resistant genes

To identify the key genes essential for endostatin‐inhibited cell proliferation, we transduced HUVEC‐flag‐SV40 cells with the genome‐scale CRISPR knockout (GeCKOv.2) library. The CRISPR/Cas9 GeCKOv.2 library contains approximately 120,000 sgRNAs that target 19,050 protein‐coding genes (six sgRNAs per gene) and 1864 microRNAs (four sgRNAs per microRNA), and also includes around 1000 “nontargeting” control sgRNAs. We transduced cells with the knockout lentivirus library, each cell was infected with only one sgRNA lentivirus on average under the condition of multiplicity of infection (MOI) < 0.4. The gene of HUVEC‐flag‐SV40 genome was randomly knocked out by the sgRNA in the library, and the infected cells were randomly divided into three groups. One group was used as the baseline for sequencing, and the other two groups were passaged to the eighth generation with or without 100 μg/mL endostatin treatment, respectively. Genomic DNA was prepared, and deep sequencing was performed to compare the abundance of all sgRNAs in the untreated and endostatin‐treated pools of cells. If a sgRNA knocked out a gene required for endostatin‐induced cellular toxicity, selection of the cells with endostatin would have no effect on cell viability. The endostatin‐treated cells with knocked‐out endostatin‐resistant genes exhibited change in their proliferation rate and a genome distribution different from the drug‐free cell group. The three cell groups were subjected to genome high‐throughput sequencing analysis to compare the enrichment of sgRNA. Among the genes whose loss conferred sensitive to endostatin, NPEPPS had the highest gene score, the second‐best scoring gene from the screen was UTS2 (Figure 1C). The results identified NPEPPS and UTS2 as potential endostatin‐resistant genes (Figure 1D).

### 2.3 Construction of highly endostatin‐sensitive cell lines

**FIGURE 2:**
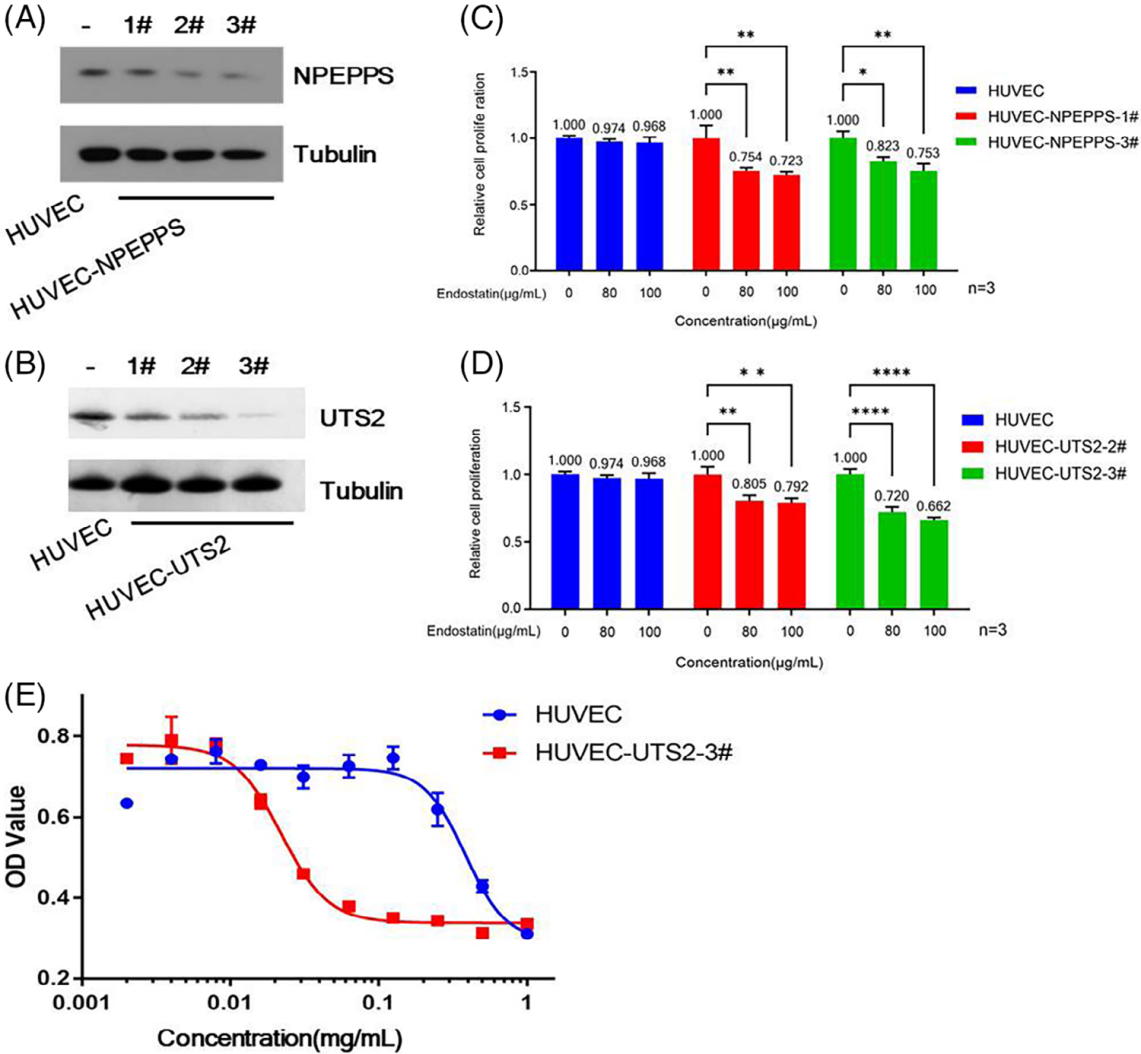
The construction of endostatin‐sensitive cell lines by knocking down NPEPPS and UTS2 genes. The decrease of the expression of the NPEPPS (**A**) or UTS2 (**B**) gene was detected by Western blot, using tubulin as the control protein. Lower concentrations of endostatin affected the relative proliferation rate of NPEPPS (**C**) or UTS2 (**D**) knockdown cell lines, where the duration of drug action was 3 days (*p < 0.05; **p < 0.01; ****p < 0.0001). (**E**) The endostatin dose–response curves of human umbilical vein endothelial cell (HUVEC)‐UTS2‐3# cells and primary HUVEC cells were compared, fitting the data points with four parameters.

For the HPEPPS and UTS2 genes, three targets were redesigned. By performing gene knockdown in HUVEC‐flag‐SV40 cells in accordance with RNA interference principle, six new cell lines were successfully constructed, namely, HUVEC‐HPEPPS‐1#/2#/3# and HUVEC‐UTS2‐1#/2#/3#. The decrease of the expression of the NPEPPS or UTS2 gene to varying degrees was detected in the transformed cells (Figure 2A,B). In drug sensitivity experiments, the cell lines with gene knockdown exhibited a reduction in proliferative activity at lower drug concentrations (80–100 μg/mL), implying an increased sensitivity to endostatin. The comparison of the degree of decline in proliferative activity showed the highest drug sensitivity in HUVEC‐UTS2‐3# cells (Figure 2C,D), in which the effective endostatin concentration was decreased from 1 mg/mL to 0.1 mg/mL with a good dose–response curve (Figure 2E). These results corroborated the successful construction of a cell line with high endostatin sensitivity. The biological activity assay method of endostatin will be established based on HUVEC‐UTS2‐3# cells in the future.

### 2.4 The optimization of the detection method of endostatin's biological activity using HUVEC‐UTS2‐3# cells

**FIGURE 3:**
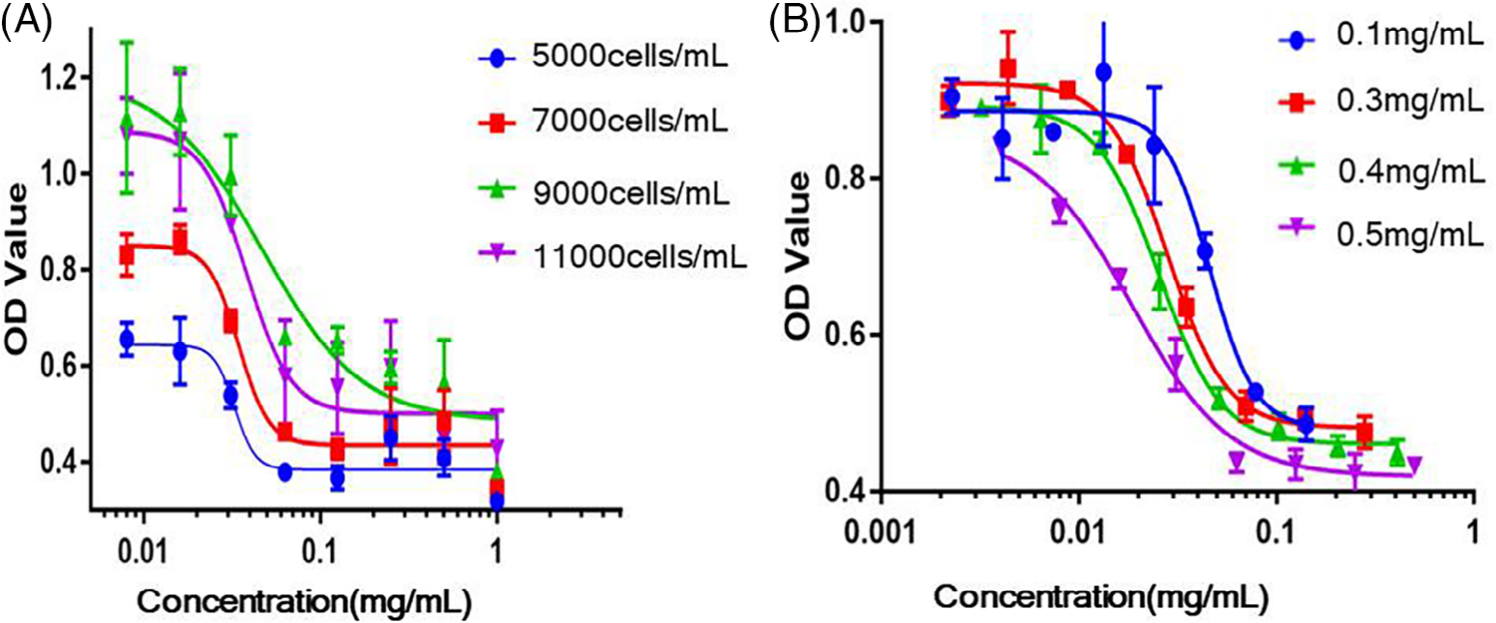
The optimization of the method for detecting the biological activity of endostatin based on human umbilical vein endothelial cell (HUVEC)‐UTS2‐3# cells. (**A**) The drug action time and predilution concentration were fixed, and the dilution ratio was two times. The initial cell density of the gradient was set. The measured data results were compared, and three parallels were set for each data. (**B**) The initial cell density was set to 9000 cells/mL. The predilution concentration of the gradient was set, and the dilution ratio was two times. The measured data results were compared, and three parallels were set for each data.

The biological activity of endostatin was determined using HUVEC‐UTS2‐3 # as the detection cell to optimize the method. The experiment was conducted by setting 96 h drug action time, 1 mg/mL prediluted drug concentration, and a gradient cell density. According to the signal‐to‐noise ratio (SNR) results, the optimal cell density was 9000 cells/mL (Figure 3A). This cell density was utilized to set the gradient endostatin prediluted initial concentration. The experimental test was conducted at doubling dilution. The results showed that the optimal prediluted initial concentration of endostatin was 0.2 mg/mL (Figure 3B).

### 2.5 Methodology validation

#### 2.5.1 Specificity

**FIGURE 4:**
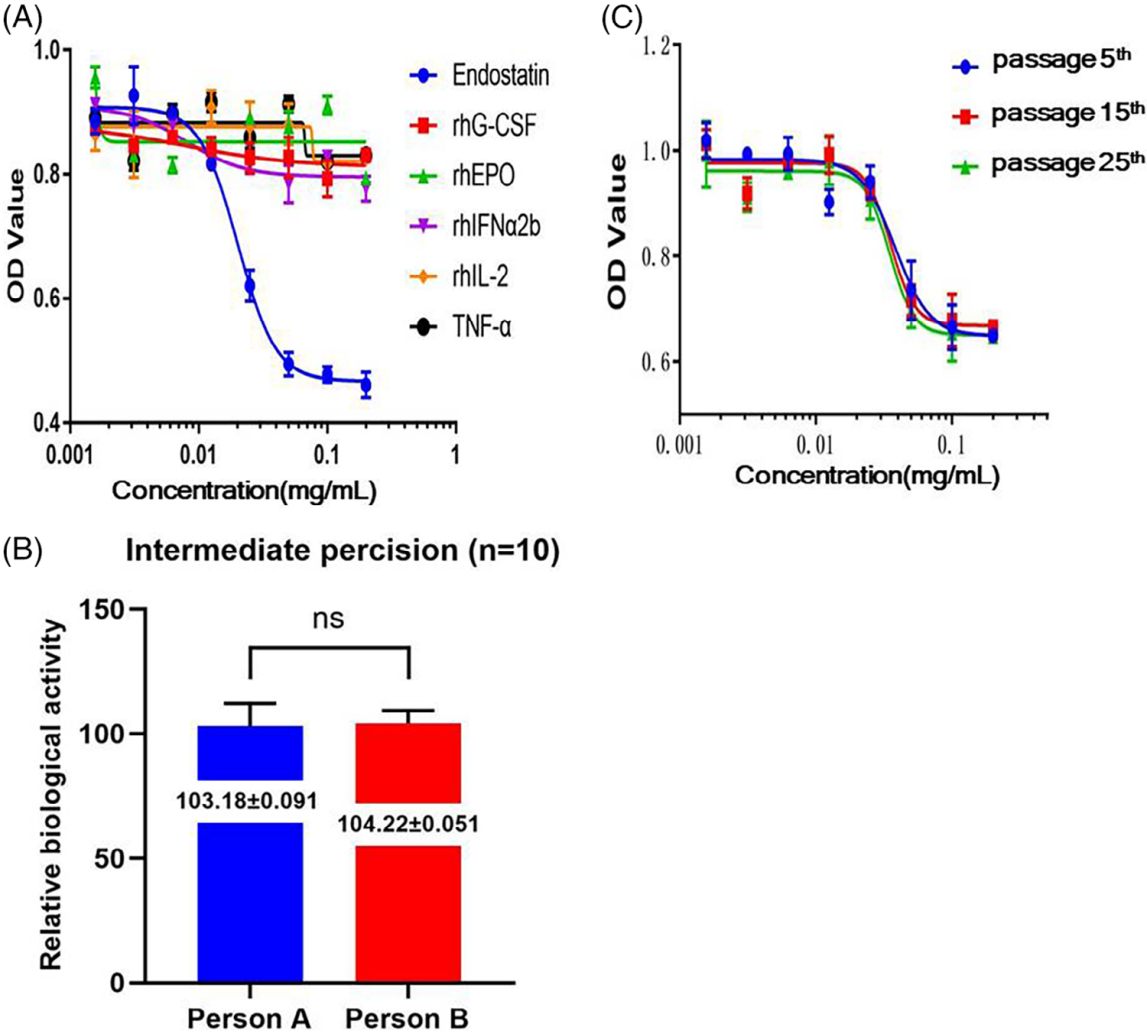
The results of the methodological validation for the determination of the biological activity of endostatin using human umbilical vein endothelial cell (HUVEC)‐UTS2‐3# as the detection cells. (**A**) Specificity validation. Endostatin was replaced with other recombinant drugs while keeping other experimental conditions unchanged, indicating the drug specificity of HUVEC‐UTS2‐3#. (**B**) The comparison of the measurement data from different experimentalists was used to analyze the intermediate precision of the method, *n* = 10, *p *= 0.4784 (ns, no significant difference). (**C**) The analysis of the stability of passage of HUVEC‐UTS2‐3# cells. The measured data curves of the biological activity of endostatin detected for the 5th‐, 15th‐, and 25th‐generation cells were compared.

A methodology validation was conducted on the newly established biological activity assay method for endostatin based on HUVEC‐UTS2‐3# cells. First, endostatin was replaced with a variety of other recombinant protein drugs for the same detection experiment. An unchanged growth activity of HUVEC‐UTS2‐3# cells indicated a good specificity of the method (Figure 4A). Although recombinant protein drugs are pure single proteins, the components in gene therapy drugs are relatively complex, and there may be multiple cytokines in active drug ingredient. The newly established biological activity assay method may achieve specific detection of biological activity of endostatin among other cytokines.

#### 2.5.2 Accuracy

Taking the biological activity test results of endostatin samples at the concentration of 0.2 mg/mL (3.2 × 10^3^ U/mL) as the theoretical value, the biological activity of endostatin was detected in the same batch exploiting the new method at different predilution concentrations. The recovery rate of the solutions with theoretical titers of 1.6 × 10^3^, 2.4 × 10^3^, 3.2 × 10^3^, 4.0 × 10^3^, and 4.8 × 10^3^ U/mL was 95.73%, 103.97%, 99.84%, 94.36%, and 103.97%, respectively. The average recovery rate was 99.57% and the relative standard deviation (RSD) < 15%, indicating a good accuracy of the method (Table 1).

**TABLE 1:** The experimental results of the accuracy of the biological activity of endostatin‐sensitive cells (*n* = 3).

Theoretical potency (U/mL)	Relative potency (U/mL)	Recovery (%)
1.6 × 10^3^	1.53 × 10^3^	95.73
2.4 × 10^3^	2.50 × 10^3^	103.97
3.2 × 10^3^	3.19 × 10^3^	99.84
4.0 × 10^3^	3.77 × 10^3^	94.36
4.8 × 10^3^	4.99 × 10^3^	103.97
Average	–	99.57
RSD	–	4.5

#### 2.5.3 Precision and reproducibility

Taking the biological activity test results of endostatin samples at the concentration of 0.2 mg/mL (3.2 × 10^3^ U/mL) as the theoretical value, the new assay method was repeated thrice a day for four consecutive days for a batch of endostatin samples at the concentration of 0.2 mg/mL. The comparison results showed 2.12%–7.67% and 8.71% coefficient of variation (CV) values of the intraday and interday precision, respectively. Both values, having RSD <15.00%, indicated a good precision (Table 2). In addition, two different operators were arranged to conduct 10 experiments employing the new method of determining endostatin's biological activity and using HUVEC‐UTS2‐3# as the detection cells at different times and different experimental sites. The results varied insignificantly between the two operators (*p* = 0.4784), implying a good reproducibility of the new method (Figure 4B).

**TABLE 2:** The experimental results of the precision of the biological activity of endostatin‐sensitive cells (*n* = 3).

Days	Relative potency (U/mL)			Mean value	Intraday CV (%)
Day 1	3.39 × 10^3^	3.26 × 10^3^	3.35 × 10^3^	3.33 × 10^3^	2.12
Day 2	3.34 × 10^3^	3.49 × 10^3^	3.69 × 10^3^	3.51 × 10^3^	5.00
Day 3	2.92 × 10^3^	3.05 × 10^3^	3.05 × 10^3^	3.01 × 10^3^	2.43
Day 4	2.83 × 10^3^	3.19 × 10^3^	2.77 × 10^3^	2.93 × 10^3^	7.67
Mean value	3.19 × 10^3^				
Interday CV (%)	8.71				

#### 2.5.4 Stability of passage

The HUVEC‐UTS2‐3# cells were passaged to the 25th generation, and the biological activity of endostatin was tested on the 5th‐, 15th‐, and 25th‐generation cells. The curves of the measurement results were consistent (Figure 4C) without significant differences. This result indicated that the growth status of the cells remained stable within 25 generations and met the detection requirements.

### 2.6 Agreement between new and original methods for the detection of endostatin activity

**FIGURE 5:**
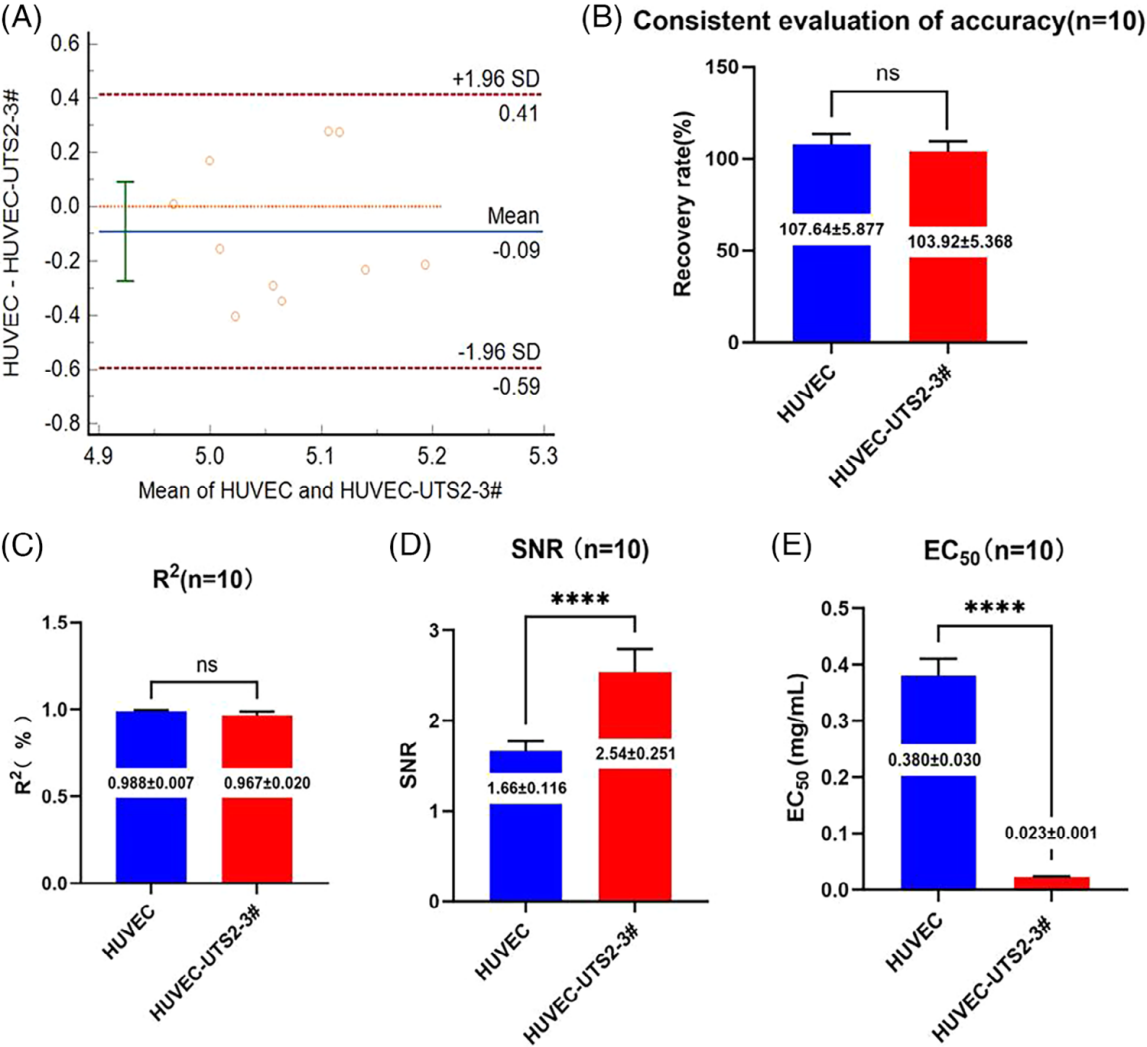
The comparison of new and original methods for the detection of the endostatin activity. (**A**) Compared new with original methods in detective values. Each plot represents the ratio of new versus original assay. The upper and lower dotted lines define the agreement limits within which 95% of differences between the two methods are expected to lie, and the middle dotted line represents the average ratio of new versus original assay (*p* = 0.2926). The comparison of the recovery rate of the detection of the potency of 50% active samples (*n* = 10, *p *= 0.1148) (**B**), four‐parameter fitting *R* ^2^ (**C**), EC_50_ values (*n* = 10) (**D**), and signal‐to‐noise ratio (SNR) (*n* = 10) (**E**) of the new and original methods (ns, no significant difference; ****p < 0.0001).

The endostatin sample was concentrated to 7.5 mg/mL and divided into 10 parts. Then, the activity of each endostatin sample was detected using primary HUVEC cells and HUVEC‐UTS2‐3#cells. The Bland–Altman method was used to analyze and compare the detection results of the two methods. An insignificant difference between the results of two methods (*p *= 0.2926, Figure 5A,B) and 95% confidence interval (CI) of the detection concentration values of all samples implied that the measurement method for endostatin's biological activity using HUVEC‐UTS2‐3# as the detection cell was consistent with the original method (Figure 5A,B).

In the detection of the biological activity of recombinant drugs, four‐parameter fitting model, EC_50_ (50% effective concentration), and SNR are important experimental indicators. The experimental data of the new and original detection methods indicated that the four‐parameter fitting *R* ^2^ of the HUVEC‐UTS2‐3# cells–based endostatin activity determination method did not differ significantly from the original method (0.988 and 0.967, respectively, *p *> 0.05, Figure 5C). The EC_50_ of the new method was 0.023 mg/mL, which was about 10 times lower than that of the original method (0.38 mg/mL) (*p* < 0.05) (Figure 5D). This difference accentuated that the new method improved the sensitivity of endostatin activity detection. Moreover, the new method exhibited a significantly higher average SNR (2.54) than the original method (1.66; *p* < 0.05) (Figure 5E). Overall, the results highlighted the superiority of new method over the original one.

## 3 DISCUSSION

Nowadays, the use of recombinant protein drugs is substantially increasing in clinical practice. Thus, the biological activity detection methods are extremely important for the quality control of biological drugs.^26^ At present, the reporter gene method can be used to solve the biological activity detection of recombinant proteins that are involved in a single signaling pathway or have no interference between signaling pathways. However, there is no suitable method to solve the biological activity detection of recombinant proteins that are involved in complex or unknown signaling pathways. The biological activity of recombinant human endostatin is particularly difficult to be determined. At present, endostatin's biological activity is assessed by using the HUVEC cell growth inhibition method. This method has poor cell reactivity and requires high drug concentration, which makes it impossible to prepare predilution of the drug. Consequently, the high protein and excipient concentration can possibly exert an impact on the detection results. The experimental time of this method is also relatively long, and the temperature is high during the process; these may deteriorate the drug, eventually affecting the experimental results. Moreover, HUVEC cells are primary cultured cells and cannot be passaged for more than 10 generations. Their instability can result in uncertain detection results. In order to solve the above problems, the most direct strategy is to increase the cell sensitivity and reduce the effective drug concentration. In this study, HUVEC cells were first immortalized, and then the endostatin‐resistant genes were screened utilizing the CRISPR/Cas9 knockout library containing 20,000 genes and by exploiting CRISPR/Cas9 gene editing technology. Subsequently, immortalized HUVEC were genetically modified to obtain cell lines with high sensitivity to endostatin. Finally, based on the sensitive cell lines, a new method for measuring endostatin's biological activity was established. The methodological validation results met the requirements for activity testing. The detection results of the new and original methods were consistent, and the new method was more sensitive and stable.

In this study, a plasmid vector containing the SV40 sequence was packaged into lentivirus and utilized to infect primary HUVEC cells. The SV40 fragment was inserted into its genome to obtain the immortalized cell line HUVEC‐flag‐SV40. Commercial CRISPR/Cas9 gene knockout libraries are often packaged as lentivirus to infect cells with high efficiency.^27^ The CRISPR/Cas9 gene knockout lentivirus library was used to infect the immortalized HUVEC‐flag‐SV40 cells. To ensure that each HUVEC‐flag‐SV40 cell was only infected with one specific sgRNA, we chose the MOI of 0.4 for lentivirus infection.^25^ This could prevent any interference with the screening of drug‐resistant genes and avoid overloading a single cell with excessive lentivirus, which could affect analysis and validation. Additionally, this approach was adopted to ensure that a sufficient number of cells were infected with the lentivirus. After lentivirus library infected the HUVEC‐UTS2‐3# cells, most of the cells were entered one gRNA for each as the MOI was 0.4, so the mRNA changes after knocking out multiple genes is not clear. This means that sensitive cells may not the most sensitive, and there is still a possibility of improvement. After the lentivirus infection, the cells were divided into dosing and control groups and cultured continuously to enrich drug‐resistant genes. Gene sequencing was performed on the dosing and control groups, and two potential endostatin resistance genes, NPEPPS and UTS2, were finally screened out. Although the sensitivity was poor, endostatin inhibited the HUVEC cell growth. We first chose to knockdown the expression of the resistant genes in the immortalized HUVEC cell lines to obtain the endostatin‐sensitive immortalized HUVEC cell lines. Three targets were selected for each gene. A gene knockdown plasmid was constructed and packaged by lentivirus to infect HUVEC‐flag‐SV40 cells. After verification by CCK‐8, the HUVEC‐UTS2‐3# cell line showed the highest sensitivity to endostatin with a 10 times reduced effective drug concentration to about 0.1 mg/mL. This substantial reduction in effective drug concentration can enable predilution of endostatin samples, thus reducing the influence of excipients and high protein concentration on the test results.

This study established a new method for measuring the biological activity of endostatin based on HUVEC‐UTS2‐3# cells and conducted methodological validation. First, the predilution ratio, number of cells, and doubling the dilution of the original method were optimized, and a new method for measuring endostatin activity was established. The accuracy, precision, specificity, and other indicators of the newly established activity determination method were verified. The new method demonstrated less than 15% RSD value of the accuracy recovery rate and CV values of 2.12%–7.67%, and 8.71% for intra‐ and interday precisions, respectively. This result is in accordance with the acceptance criteria for CV values specified in the AAPS/FDA seminar, that is, ≤15%–20%. Therefore, the precision of the new method met the standards. The comparison between the new and original methods showed that the detection results and accuracy were consistent. The new method had a lower EC_50_ value, indicating higher sensitivity, and a significantly higher SNR, which means that the data obtained were more accurate and reliable than that of the original method.

The dose–response curve of the new method for activity detection based on HUVEC‐UTS2‐3# cells met the endostatin biological activity detection requirements, highlighting its potential to replace the original method completely. Our future research plan includes validating the new method for products from different manufacturers and batches and conducting a joint validation with other laboratories to prepare alternative methods.

In conclusion, this study successfully used the CRISPR/Cas9 knockout library to screen potential endostatin‐resistant genes, constructed endostatin‐sensitive cell lines, and successfully used these cell lines to establish a new detection method for endostatin's biological activity. In this study, in the case of complex or unknown signaling pathways, a stable and sensitive cell line for activity detection was obtained by transforming the primary effector cells. Following the reporter gene method,^3^, ^4^, ^5^, ^6^, ^7^ a new gene‐editing method route was created for the application of transgenic cell method to study the biological activity of recombinant protein drugs. The established method can overcome the problems related to the intricate measurement of the activity of recombinant protein drugs.

## 4 MATERIALS AND METHODS

### 4.1 Sources of cells and the main reagents

The competent *Escherichia coli* DH5α, plasmid pCDH‐flag, primary HUVEC cells, HEK293T cells, and the endostatin standard substance were all preserved in our laboratory.

### 4.2 Immortalized transformation of HUVEC cells

Large T antigen SV40‐transfected and immortalized primary HUVEC cells were accomplished. The SV40 fragment was amplified by polymerase chain reaction (PCR) (F: GAAGATCTGATCAAACTCTGTCTTTG; R: ATAAGAATGCGGCCGCTGCTTGTTTCTTCAATAA), ligated to the plasmid pCDH‐flag by enzyme digestion, and the plasmid pCDH‐flag‐SV40 was obtained after sequencing. Plasmid psPAX2 (Addgene, Cat. No. 12260), plasmid pMD2G (Addgene, Cat. No. 12259), and plasmid pCDH‐flag‐SV40 were cotransfected into HEK293T cells (cultured with dulbecco’s modified eagle‘s medium (DMEM) containing 10% fetal bovine serum (FBS), and SV40 was packaged for lentivirus infection. Primary HUVEC cells (cultured with endothelial cell medium (ECM) containing 5% FBS and 1% endothelial cell growth supplement) were then infected with lentivirus, and monoclonal cells were selected and passed to the 10th generation in ECM medium (ScienCell Research Laboratories, Cat. No. 1001). The surviving cells were immortalized HUVEC cells, named HUVEC‐flag‐SV40, and the expression of large T antigen was detected.

### 4.3 The screening of endostatin‐sensitive genes by CRISPR/Cas9 library

Gene editing was afforded randomly for the immortalized HUVEC cells exploiting a CRISPR/Cas9 gene knockout library, and the potential drug resistance genes were screened.^28^ First, Lipofectamine 3000 (Invitrogen, Cat. No. L3000015) was used as the transfection reagent. Then, plasmid psPAX2, plasmid pMD2G, Human CRISPR Knockout Pooled Library (GeCKO v2), plasmid library A (Addgene, Cat. No. 1000000048), and plasmid library B (Addgene, Cat. No. 1000000049) were cotransfected into HEK293T cells to package and obtain a gene random knockout lentivirus library.

The HUVEC‐flag‐SV40 cells were subsequently amplified, cultured, and inoculated into 75 flasks (3 × 10^6^ cells/flask) of 175 cm^2^ Petri dishes. When the cells grew to about 40% coverage, the medium was replaced with a 10 mL ECM medium containing 10 μg/mL polybrene (Santa Cruz, Cat. No. sc‐134220). After 30 min, a mixture of 12 mL lentivirus solution and ECM medium was added to make MOI = 0.4, and to ensure that most cells get infected with only one sgRNA. Following 48 h, the infected cells were screened for resistance over 3 days by utilizing puromycin (2 μg/mL)‐containing ECM medium. The surviving cells were collected, counted, and divided into three groups; each group comprising approximately 6 × 10^7^ cells. The cells of first group, namely, HUVEC P0, were washed twice with phosphate buffer and stored under liquid nitrogen. The second cell group, namely, dosing group, was inoculated in 20 new cell culture bottles (3 × 10^6^ cells/bottle) and added with endostatin‐containing medium (final concentration for 100 μg/mL). The third cell group (control) was processed analogous to the drug group with exception of being cultured in a drug‐free medium. The dosing and the control groups were counted and passaged; the number of cells was ≤1.2 × 10^8^ before each passage. During the passage, the number of cells in each bottle should be 6.0 × 10^7^, and the cells in the two groups needed to be screened for eight generations. Finally, RNA‐sequencing was performed on the eighth generation of the dosing (Endo P8) and control (HUVEC P8) groups, and the frozen HUVEC P0 cells to analyze the enriched sgRNA and corresponding genes (Figure 1C).

### 4.4 Genome high‐throughput sequencing analysis

The sgRNA sequences and corresponding target genes were analyzed using the data of CRISPR gene knockout plasmid library A and plasmid library B. All cell groups were subjected to genome high‐throughput sequencing. The MAGeCK‐RRA algorithm was used to assign scores to the robust rank aggregation (RRA) of the corresponding gene sites according to the enrichment of sgRNA in the sequencing results. Taking the results of the HUVEC P0 group as a reference, the lower ratio of the experimental group to the control group indicated the higher gene sensitivity to the drug. Through negative screening, the screening indicators were selected as lfc (log3 fold change) and FDR (adjusted *p* value), referencing log3 fold change <−2 and FDR <0.05.

### 4.5 NPEPPS and UTS2 gene knockdown

Three knockdown targets were designed for NPEPPS and UTS2 genes each (the three targets of NPEPPS were NPEPPS(H)‐1: GCATTTGTTGTGGGTGAAT, NPEPPS(H)‐2: GCCCATCAATGGTTTGGAA, and NPEPPS(H)‐3: GCTTCCCAGATTGATT. The targets of UTS2 included UTS2(H)‐1: GCTAGAAAGAGCTTCCCTT, UTS2(H)‐2: GCCAGAATCTGGAAACCAT, and UTS2(H)‐3: GGAGGAGCTAGAAAGAGCT). Three NPEPPS and three UTS2 gene knockdown plasmids were, respectively, constructed on the plasmid JLUV6‐GFP‐Kan‐Puro (JTS, Cat. No. C02001) using the corresponding shRNA. The gene knockdown plasmid, plasmid psPAX2, and plasmid pMD2G were transfected into 293T cells, and the gene knockdown lentivirus was packaged. HUVEC‐flag‐SV40 cells were infected with the gene knockdown lentivirus and screened in ECM medium containing 2 μg/mL puromycin for 48 h after infection. The surviving cells were identified as NPEPPS or UTS2 gene knockdown cells after monoclonal selection. The expression of the target gene was decreased under the effect of RNA interference, and the expression was detected by western blot.

### 4.6 Western blot

Cells were collected, washed with phosphate buffer, and then lysed utilizing radio immunoprecipitation assay (RIPA) lysis buffer (Applygen, Cat. No. C1053). Then, protein loading buffer was introduced into the supernatant and denatured at 95°C. The electrophoresis was performed using NuPAGE 4%–12% Bis‐Tris Gel (Invitrogen, Cat. No. NP0321BOX) at 120 V, followed by transfer to polyvinylidene fluoride (PVDF) membrane, which was then blocked and incubated at room temperature for 1 h. The first antibody hybridization was performed at 4°C overnight, while the secondary antibody hybridization was performed at room temperature for 30 min after washing with Tris‐buffered saline + tween (TBST) three times. Among them, tubulin antibody (Abcam, Cat. No. ab7291) was diluted at 1:500, while its secondary antibody (haptoglobin related protein (HPR) antimouse antibody, Abcam, Cat. No. ab6728) was diluted at 1:2000. The dilutions of flag protein primary (Abcam, Cat. No. ab213519) and secondary (HPR antimouse antibody, Abcam, Cat. No. ab6728) antibodies were diluted at 1:2000 and 1:5000, respectively. NPEPPS antibody (Abcam, Cat. No. ab77643) was diluted at 1:4000, and its secondary antibody (horseradish peroxidase (HRP)‐labeled antisheep antibody, Abcam, Cat. No. ab6741) was diluted at 1:2000. Similarly, UTS2 antibody (Abcam, Cat. No. ab48609) and its secondary antibody (HRP‐labeled antirabbit antibody, Abcam, Cat. No. ab6721) were diluted at 1:4000 and 1:2000, respectively. The color was developed with ECL luminescence solution following a triplicate wash with TBST.

### 4.7 The inhibitory rate of endostatin on cells detected by CCK‐8

Endostatin samples were set at three final concentration points, namely, 0, 80, and 100 μg/mL. The same amount of primary HUVEC and HUVEC cell lines knocked down by drug resistance gene were added at 50 μL/well, making the total volume 200 μL and setting three parallel wells for each concentration gradient. The samples were incubated at 37°C, 5% CO_2_ for 3 days. Subsequently, 20 μL of CCK‐8 solution (Dojindo Laboratories, Cat. No. CK04‐1000T) was introduced to each well and incubated under CO_2_ for 2 h. Finally, the absorbance was measured at a wavelength of 450 nm using a microplate reader.

### 4.8 Detection of endostatin biological activity based on primary HUVEC cells

The primary HUVEC cells were inoculated into a 96‐well plate using ECM complete culture medium with 5000 cells/mL and 160 μL cell suspension per well and placed in a 5% CO_2_ incubator at 37°C. Then, 40 μL of a twofold diluted endostatin sample (an initial concentration of 5 mg/mL) was added to each well. A total of eight concentration gradients, with three parallel wells for each, were set up, and the culture was continued in the incubator for 96 h. Afterward, 20 μL of CCK‐8 solution was added to each well, and incubated at 37°C, 5% CO_2_ for 2 h. Subsequently, the cells were mixed well and their optical density (OD) value was determined at a wavelength of 450 nm using a microplate reader. SoftMax Pro software was used to scan and record data. The biological activity of endostatin was calculated using a four‐parameter equation.

### 4.9 Detection of endostatin biological activity based on HUVEC‐UTS2‐3# cells

The HUVEC‐UTS2‐3# cells were inoculated into a 96‐well plate using ECM complete culture medium with 9000 cells/mL at 160 μL cell suspension per well and kept in an incubator at 37°C under 5% CO_2_. Endostatin was diluted to 1.0 mg/mL from its initial reaction concentration of 0.2 mg/mL. There were 8 concentration gradients in total, each having 3 parallel wells. The wells were then exposed to corresponding drug dilutions and cultured for 96 h in an incubator (37°C, 5% CO_2_). Subsequently, each well plate was added with 20 μL of CCK‐8 solution and incubated in a CO_2_ incubator for 2 h. Using a microplate reader, the OD value was then determined at a wavelength of 450 nm. Data were scanned and recorded by employing SoftMax Pro software, and the biological activity of endostatin was calculated using a four‐parameter equation. To verify the specificity of HUVEC‐UTS2‐3# cells to endostatin, the endostatin drug in this method was replaced with recombinant human interleukin‐2, recombinant human granulocyte colony‐stimulating factor, recombinant human erythropoietin, recombinant human interferon α2b, and recombinant human basic fibroblast growth factor. The solution was twofold serial dilution of the initial reaction concentration of 0.2 mg/mL, there were eight concentration gradients in total, each having three parallel wells.

### 4.10 Bland–Altman analysis

Bland–Altman is considered the standard method for assessment of agreement between two methods of measurement.^29^ Bland–Altman can calculate the limits of agreement between two measurement results and visually reflect this consistency limit using graphical methods. The usage of this method is also to demonstrate that there is no difference in the results between the measurements of the newly method and the original method. The Bland–Altman method was adopted to evaluate the consistency range of the two measurement results. A diagram was drawn to visually reflect the distribution of the consistency range and the measurement gap between the two methods. The upper and lower red lines represented the upper and lower limits of the 95% CI of the curve, and the middle blue line depicted the average differences.

### 4.11 Statistical analysis

SoftMax Pro v5.4 and GraphPad Prism 8.0 were used for data processing and statistical analysis. Student's *t*‐test was used for comparison between two groups, and four‐parameter fitting was used for function fitting of data. A value of *p *< 0.05 was considered statistically significant.

## AUTHOR CONTRIBUTIONS

Conceptualization, design of the study, and revised the manuscript: Junzhi Wang and Shanhu Li. Acquisition of data, or analysis and interpretation of data: Xi Qin, Yifang An, Xiang Li, Fang Huang, Dening Pei, and Hua Bi. Drafting the paper or revising it critically for important intellectual content: Yong Zhou, Xinchang Shi, Wenhong Fan, Youxue Ding, and Shuang Li. All the authors have read and approved the final manuscript.

## CONFLICT OF INTEREST STATEMENT

Shuang Li is an employee in Sinovac Research & Development Co., but has no potential relevant financial or nonfinancial interests to disclose. The other authors have no conflicts of interest to declare.

## ETHICS STATEMENT

Primary HUVEC cell was purchased from ScienCell Research Laboratories (America), Cat. No. 8000. This study does not involve any ethical issues.

## Data Availability

The data included in this study are available upon request from the corresponding author.
